# The Oxidation Behaviour and Notch Wear Formation of TiAlN Coated Tools Using Different Oxidation Techniques

**DOI:** 10.3390/ma14061330

**Published:** 2021-03-10

**Authors:** Wit Grzesik, Joanna Małecka

**Affiliations:** Faculty of Mechanical Engineering, Opole University of Technology, 45-271 Opole, Poland; w.grzesik@po.edu.pl

**Keywords:** nitride coating, diffusion couple method, oxidation wear, machining

## Abstract

This paper proposes a novel approach to assessing oxidation behavior of TiAlN coatings with defined stoichiometry on the rake and flank surfaces. This is based on the multi-parametric comparison of the oxidation effects detected on the coatings’ surfaces resulting from static diffusion couple tests. In this experimental study the diffusion couples consisting of Ti-based and Ni-based alloys and coated TiAlN cutting inserts are tested, respectively. The optimum oxidation temperature was determined by annealing the selected TiAlN coating in a high temperature chamber at temperatures: 700 °C, 800 °C, 900 °C and 1000 °C in air. Concurrently, the mass change and corresponding thickness of the Al_2_O_3_ oxidized layer were measured and computed. The comparison of oxides produced covers the surface morphologies, chemical elements and phases which were analyzed by means of SEM (scanning electron microscope), EDS (energy dispersive spectroscopy) and XRD (X-ray diffraction techniques). Additionally, scratch tests were performed to assess the penetration depth down to the substrate and coating failure mechanism after oxidation in diffusion couples. An acceptable similarity of Al_2_O_3_ films formed on the TiAlN coating surfaces in diffusion couples and machining processes was established.

## 1. Introduction

Nowadays, a strong challenge towards machinability improvement noticed in production and manufacturing areas results in the widespread application of cutting tool materials coated by multi-functional layers which can withstand high contact pressure and high temperature. These efforts are predominantly addressed to a group of difficult-to-machine aerospace materials (superalloys) which are classified within the ISO S group of engineering materials. It is expected that they should not only retain greater hot hardness and excellent resistance to slow down diffusion including oxidation resistance in atmospheric air on account of penetration air to the cutting zone in dry machining [1].

In particular, Ti_1-x_Al_x_N and Al_x_Ti_1-x_N coatings with various stoichiometric relationships of Al/Ti (x = 0.5−0.7) have versatile applications in the restricted machining operations of Ni-and Ti-based heat resistant superalloys (HRSA) [2,3]. It is very important from the practical point of view that by varying the Al content in the TiAlN coating, its mechanical and tribological properties improve due to promoting the formation of an outer Al_2_O_3_ layer during machining operations. However, the higher Al content causes a lower oxidation rate [4]. On the one hand, dual-phase and hcp (hexagonal close-packed)-structured coatings with high Al contents are more wear resistant in comparison to fcc (face-centered cubic) structured coatings [1,5,6]. The crystal structure of TiAlN is fcc when the AlN content is less than 60 mol%, whereas hcp structure occurs if the AlN content is higher than 70 mol% [5]. On the other hand, increasing the Al content above x = 0.60 results in a lower oxidation resistance of the AlTiN coating due to the precipitation of the wurtzite-type AlN [6]. A potential effect is either increasing the rate of oxygen inward diffusion or decreasing the rate of Al diffusion into the surface.

It was documented, based on the pin-on-disc tests [7], that for sliding materials with dominating severe adhesive wear, the protective function of the TiAlN coating is not sufficient. As a result, the research problem arises of how the formation of Al_2_O_3_ protective layer can be predicted for machining applications. At present, some oxidation and diffusion investigations are carried out [3,8]. They are carried out in air atmosphere or in vacuum at different times and oxidation temperatures. These conditions allow replicating crater or notch wear conditions, respectively. However, it is not clear whether the contact zones between the coating and the workpiece material are subjected to atmospheric oxygen or not. In particular, research on the oxidation behavior of cermets, uncoated cemented carbides and pCBN tools in coupling with Inconel 718, alloyed steel and hard steel and was performed in a controlled-environment chamber [2,3]. Hatt et al. [9] proposed replicating the tool crater wear in machining of (α + β) titanium alloys using the diffusion couple method in vacuum. However, only crater wear at the rake face was taken into account. Moreover, the oxidation test on uncoated and TiAlN/TiN coated carbide inserts were reported in [10,11]. They revealed the evolution of the visible notch wear (VB_N_) resulting from severe oxidation of the peripheral zone of the flank face.

Extended investigations of tool wear mechanisms when machining of Inconel 718 and Ti6Al4v alloys using a set of TiAlN coated inserts were carried out in other research studies [11,12,13]. They confirmed the predominant notch wear mechanism on the flank face in dry machining of these superalloys. In this paper, the oxidation tests were carried out at four different temperatures: 700 °C, 800 °C, 900 °C and 1000 °C with regard to the connection between TiAlN-coating and the diffusion couple interface created by the Ti6Al4V and Inconel 718 (IN 718) and this coating. After the tests were finished, the obtained data were related to tool wear data under dry machining condition and different machining times. It was reasoned that oxidation effects documented by X-ray diffraction (XRD) and EDS analysis of the oxidized coating surfaces are similar for the static diffusion-couples and dynamic tool wear tests.

## 2. Experimental Details and Measurements

In this experimental study, two aerospace heat resistant alloys (HRSA) including Ti-6Al-4V alloy (Bimotech, Wroclaw, Poland) with typical two-phase α + β structure (hardness of 36 HRC) and nickel-based superalloy of Inconel (IN) 718 (Bimotech, Wroclaw, Poland) (brand name PWA 1469-4, hardness is 36 HRC) are used. Cutting tools coated with PVD (Physical Vapour Deposition) TiAlN (atomic ratio Ti:Al of 0.45:0.55) layer with coating thickness of about 3 μm were selected. They were a KC5010 rhombic shaped cutting inserts (Kennametal, Poznan, Poland). For machining trials, cutting speed v_c_ = 200 m/min, constant depth of cut a_p_ = 0.25 mm and feed rate of f = 0.1 mm/rev were selected.

Two short machining trials of 0.5 and 2 min were carried out for the two alloys in order to expose the oxidized Al_2_O_3_ layer on the rake and flank faces and to show the development of notch wear which is predominantly caused by ambient air penetrating from the periphery of the flank face [1,11].

In this experimental study, three different oxidation techniques were used: classical oxidation test in a free contact with air in the furnace chamber, static diffusion-couple test performed in the same chamber and dynamic diffusion-couple test under real machining conditions.

**Figure 1:**
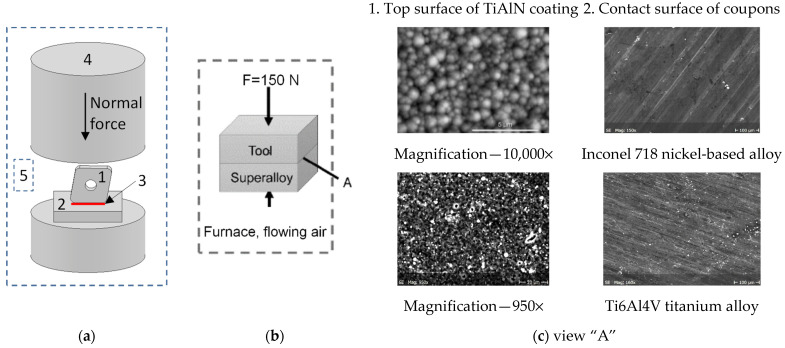
Schematic setup of static diffusion couple test (**a**) test conditions; (**b**) contact surfaces; (**c**) 1, cutting insert; 2, Inconel 718/Ti6Al4V coupons; 3, diffusion bond; 4, ceramic mass; 5, furnace with flowing oxygen; A, interface of diffusion couple.

The oxidation experiments for the TiAlN-coated inserts were carried out in air at four different temperatures: 700 °C, 800 °C, 900 °C and 1000 °C and for three annealing times of 2.5, 10 and 30 min. The computer-aided acquisition system was used to verify the actual test temperature. In the second stage, the diffusion couples, consisting of TiAlN-coated inserts and special coupons (see Figure 1) made of Inconel 718 and Ti6Al4V alloys were annealed at the constant temperature of 900 °C for 2.5, 10 and 30 min. In all tests, at least three samples were used and each oxidation test was repeated at least three times.

The oxidation effect was assessed by measurements of the mass before and after annealing using precision weighting with a resolution of ±0.1 mg [14]. Consequently, the thickness (*t_ol_*) of the deposited Al_2_O_3_ layer was assessed from Equation (1) and mass of the deposited Al_2_O_3_ layer by area unit (mol) was designate:

(1) $$t_{ol}=\frac{\Delta m_{ol}}{\rho A_{u}}$$

where:

- *Δm_ol_* = mass increment, mg/cm^2^
- *ρ* = density of Al_2_O_3_, 3986 kg/m^3^
- A_u_ = unit area, cm^2^.

Furthermore, the coating adhesion was tested by a scratch test using a micro scratch tester (MST Instrument) (Anton Paar, Warsaw, Poland) equipped with a diamond cone-shaped indenter (Anton Paar, Warsaw, Poland) by Rockwell with a tip radius of 100 μm and concurrently the coating thickness was measured accurately.

The static diffusion couple method was applied in order to quantify the diffusion interactions between the TiAlN coating and both Ti6Al4V and Inconel 718 samples in the form of a thin disc of about 2 mm in thickness. The contact disc surfaces were polished to a mirror finish using ultra fine abrasives and ultrasonically cleaned in isopropanol. During the test, they were pressed by a mass of 15 kg to promote strong contact and intensify chemical reactivity at high temperatures. 

After the test was finished, the coating and coupon’s surfaces were examined by scanning electron microscopy JEOL JSM 840A (JEOL Companies, Tokyo, Japan) and were also subjected to energy-dispersive spectroscopy X-ray microanalysis (EDS) (JEOL Companies, Tokyo, Japan) in terms of diffusion processes and oxidation effects. Phase identification of the oxide layers was performed by X-ray diffraction (XRD) (X’Pert PRO PANalytical) (Panalytical Companies, Malvern, United Kingdom). 

Figure 1 presents the setup of diffusion couple test in which the commercial cutting tool insert (1) is coupled with a disc-shaped material (2) (Figure 1a). As a result, the diffusion at the couple interface is developed correspondingly to the contact conditions created by the normal load of 150 N on the flank face (Figure 1b). The chemical compositions of the contacted materials detected by EDS analysis are specified in Table 1.

## 3. Experimental Results and Analysis

### 3.1. Oxidation Processes of TiAlN Layer Coupled with Heat Resistant Superalloys

**Figure 2:**
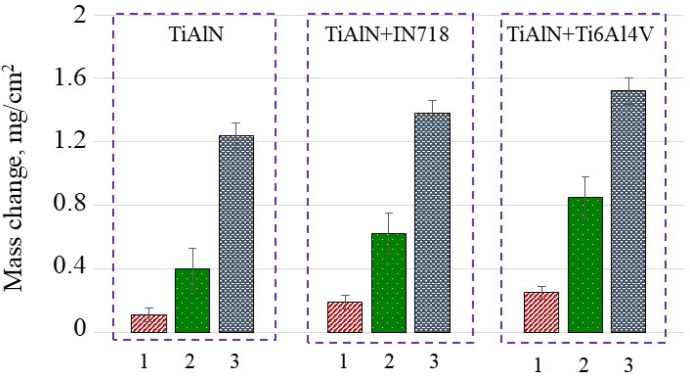
The mass gain of oxidized samples after annealing performed at 900 °C for 2.5 min (1); 10 min (2) and 30 min (3). For the diffusion couples 3 (annealing time of 30 min) the appropriate values of mass change are equal to 1.36 ± 0.06 and 1.50 ± 0.06.

The values of the mass of oxidized layers measured after annealing performed at 900 °C for 2.5, 10 and 30 min are presented in Figure 2. The first important finding is that the masses of oxidized layers deposited on TiAlN coatings in air environment and in two diffusion couples by contact with Inconel 718 and titanium Ti6-4 superalloys are visibly different. It can be noted in Figure 2 that an increase in the oxidation time from 2.5 to 30 min results in a mass increment of oxidized layer (mol) from 0.1 to 1.5 mg/cm^2^. It is seen in Figure 2 that the mass increment per area unit (oxidation rate) (*Δmol*) depends on the oxidation time and the type of oxygen access to the TiAlN coatings. The higher mass increments were measured for both diffusion couples in comparison to free oxidation by flowing air.

**Figure 3:**
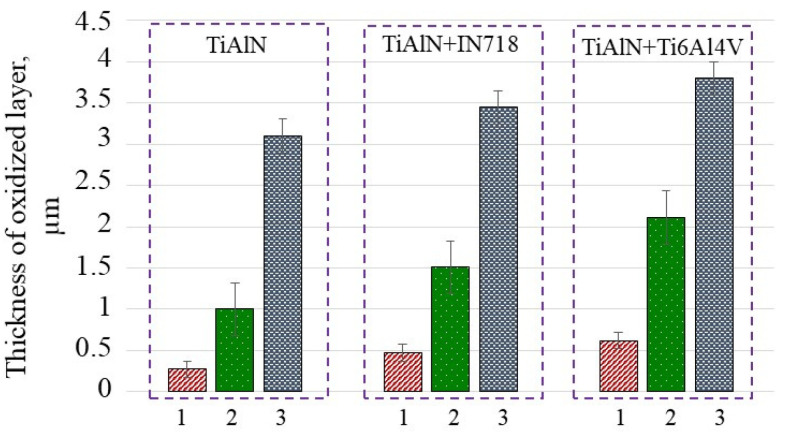
Increments of oxidized layer after annealing performed at 900 °C for 2.5 min (1); 10 min (2) and 30 min (3).

Moreover, the highest mass increment was observed for the TiAlN+Ti6Al4V diffusion couple due to additional diffusion of Ti documented by appropriate EDS analysis (for instance, see the at.% for Ti in the estimated chemical composition below). On the other hand, in the case of Ti alloys oxidized in high temperature, the mixture of Al_2_O_3_ and TiO_2_ are produced, however the Al_2_O_3_ oxide scale is thermodynamically more stable at 900 °C [15].The annealing time influences the oxidation effects in such a way that for the shortest time of 2.5 min the mass increment (bars 1 and 3 in sections for TiAlN and TiAlN + Ti6Al4V) increased by 70% and 130% in comparison to about 12% and 22% when the annealing was continued up to 30 min for diffusion couples including IN718 and Ti6-4 coupons, respectively. This is because the relation of mass increment versus oxidation time is not linear but fits a logarithmic function [13]. The corresponding values of the thicknesses of oxidized layers for different oxidation times equal to 2.5 min, 10 min and 30 min calculated using Equation (1) are presented in Figure 3.

It can be concluded based on the estimations presented in Figure 3 that the thickness of the oxidized layer increases by about three times when the annealing time increases from 2.5 to 10 min. On the other hand, the profiles of mass and thickness vs. annealing temperature shown in Figure 2 and Figure 3 satisfy the exponential function.

**Figure 4:**
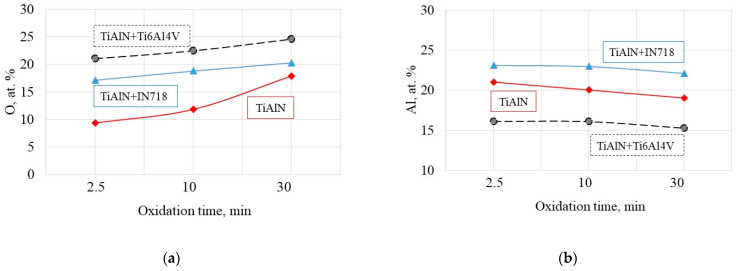

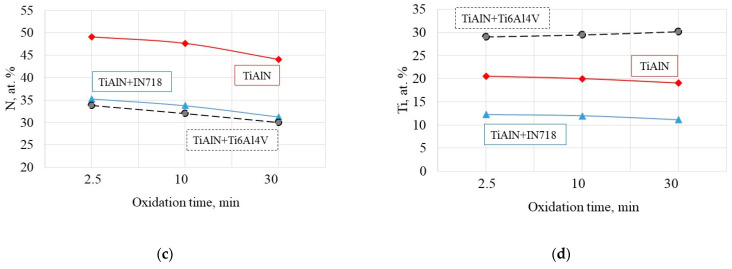
Average semi-quantitative chemical composition (estimated by EDS of oxidized layer for investigated samples: (**a**) oxygen; (**b**) aluminum; (**c**) nitrogen; (**d**) titanium. Process conditions: oxidation temperature of 900 °C, annealing times of 2.5 min, 10 min and 30 min.

The changes of chemical elements (Ti, Al, O and N at.%) determined by means of EDS analysis are specified in Table 2 and Table 3 and presented in Figure 4. The analysis of chemical contents specified in Table 2 show that the first period of oxidation was observed at 700 °C but the oxide layer of Al_2_O_3_ (about 2 μm thickness) can be formed on the TiAlN coating at temperatures about 900 °C and in about 15 min; this is in agreement with [11]. Comparatively, a similar effect occurs after 10 min annealing at 900 °C when testing the TiAlN-Ti6-4 diffusion couple (see Figure 3).

Based on the changes of chemical composition of Al_2_O_3_ layers oxidized at different temperatures (Table 2), it was observed that the content of oxygen increases up to about 30 at.% at 1000 °C, but the content of nitrogen decreases when the oxidation test is performed at higher temperatures, in other words, above 800 °C.

**Figure 5:**
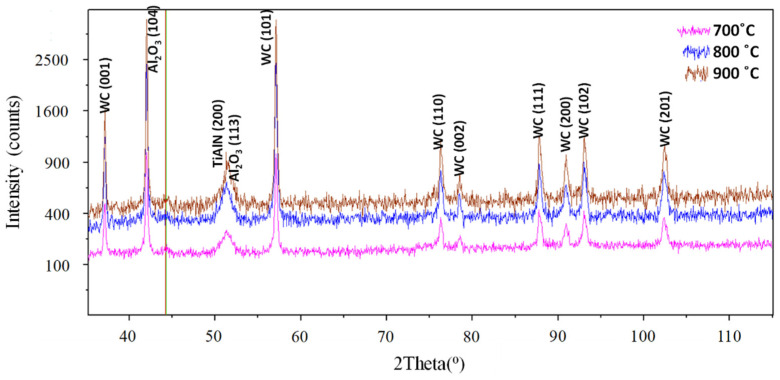
X-ray diffraction patterns of oxidation products generated at 700 °C, 800 °C and 900 °C (showing formation of Al_2_O_3_ layer over Ti_0.45_Al_0.55_N basic layer) [13].

As a result of oxygen diffusion to the TiAlN (200) coating, a stable alumina layer is formed, as evidenced by two distinct diffraction peaks on the XRD diagram (Figure 5) corresponding to the two dominant crystallographic orientations (104) and (113) (at about 2θ = 43, 8° and 51°). The Pilling–Bedworth ratio for Al_2_O_3_ is equal to PBR = 1.29 [16]. This is the criterion for the formation of a stable and dense oxidized layer. The two diffraction peaks (200) and (113) overlap.

On the other hand, for the shorter oxidation time of 5 min, which is more appropriate for the machining time, the layer thickness can be expected to be about 1 µm.

**Figure 6:**
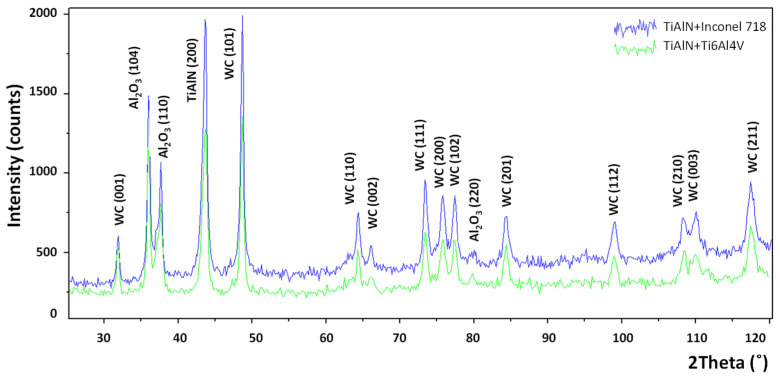
X-ray diffraction patterns of oxidation products generated in the diffusion tests at temperature of 900 °C during 15 min [13].

The XRD patterns obtained for the diffusion couples (TiAlN + Inconel 718 and TiAlN + Ti6Al4V) are shown in Figure 6. They are practically the same. It can be concluded that the Al_2_O_3_ oxide layer is formed for two different orientations of the crystals, in other words, (104) and (110) at 2θ = 38°. The more distinct peak for TiAlN (200) is detected at 2θ = 43.8°.

Based on these investigations it can be concluded that the mechanism of oxidation is practically the same for oxidation in air atmosphere and in the diffusion couple. The EDS analysis (Figure 4) showed increased concentration of Ti (30 at.%) for the couple with Ti6Al4V alloy versus about 20 at.% for the TiAlN coating and that Cr (5 at.%) and Ni (7 at.%) for the couple containing IN718 alloy. Furthermore, the content of oxygen increases when the diffusion tests with Ti6Al4V alloy are performed. This finding may explain a distinct mass gain for both diffusion couples. A more detailed explanation of this phenomenon can be made using the enthalpy analysis [9].

### 3.2. Investigation of Oxidized Coating by Scratch Test Method

In this paper, scratch tests were carried out at an linearly increasing load [17] between 30 mN–20 N at the following settings:

- the feed rate of the penetrator: 1.5 m/min,
- the length of sliding distance: 3 mm.

**Figure 7:**
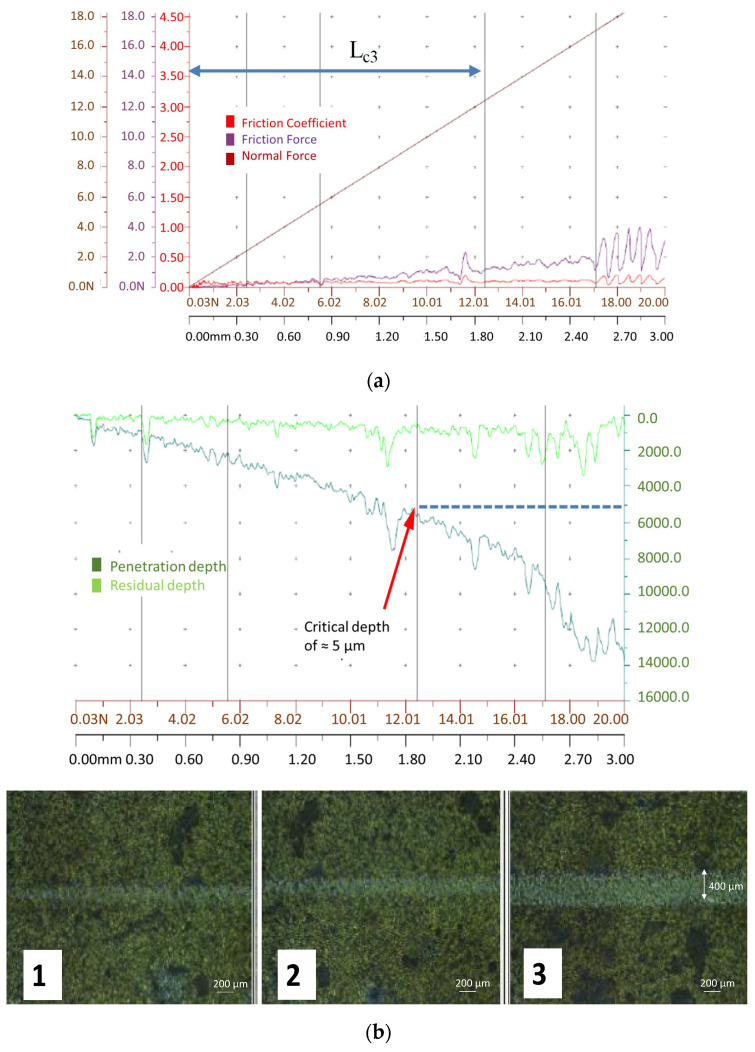
Original plots of the coefficient of friction and friction force (**a**) and optical micrographs of the scratching scars for magnification ×20 (**b**) for TiAlN samples oxidized at 900 °C for 30 min. Symbols of test stages: 1, failure initiation; 2, spalling; 3, delamination.

Simultaneously, acoustic emission signal (AE) was measured. Figure 7a,b shows the records of the friction coefficient, friction force and the penetration depth for the Al_2_O_3_ layer formed at 900 °C.

**Figure 8:**
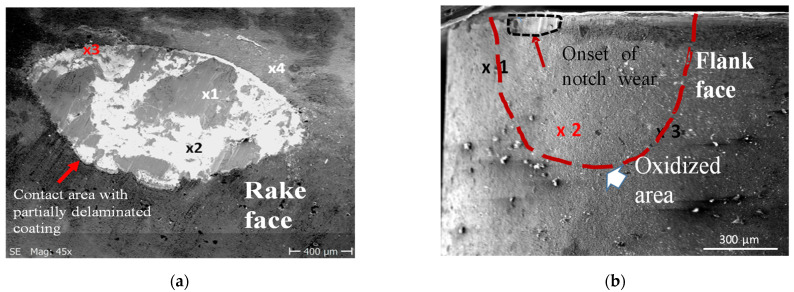
SEM images of rake (**a**) and flank (**b**) faces obtained after machining of IN718 workpiece with the cutting speed of 200 m/min; machining time of 30 s [13].

**Figure 9:**
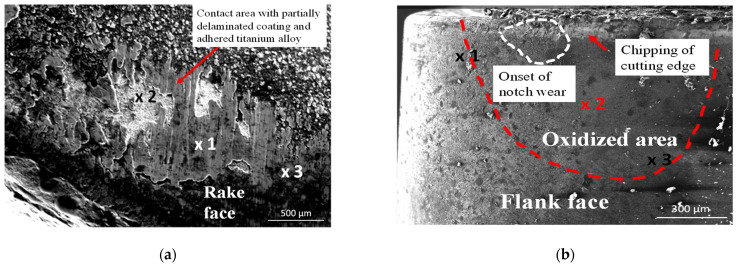
SEM images of rake (**a**) and flank (**b**) faces obtained after machining of Ti6Al4V workpiece with the cutting speed of 200 m/min; machining time of 30 s.

**Figure 10:**
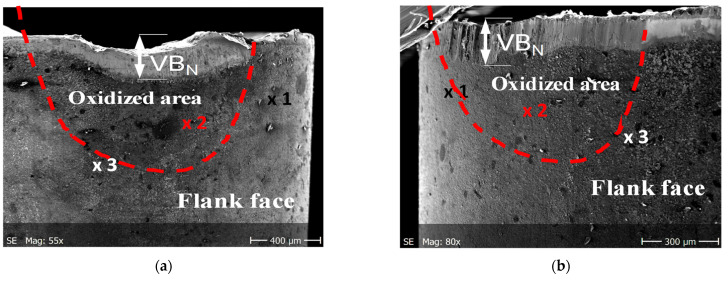
SEM images of flank faces obtained after machining of IN718 (**a**) and Ti6Al4V (**b**) workpieces with the cutting speed of 200 m/min; machining time of 2 min.

The rationale for this examination was to detect the presence of the Al_2_O_3_ oxidized layer over the basic TiAlN coating after static diffusion couple tests in order to determine the oxidation temperature (the samples were tested after oxidation at 700 °C, 800 °C and 900 °C). In contrast, scratch tests were not performed on the worn flank faces shown in Figure 8, Figure 9 and Figure 10 due to their substantial irregularities.

A low coefficient of friction in the range of 0.25 was determined at the sliding distance of about 1.82 mm (Figure 7a) and the adhesion-related critical load was equal to L_c3_ = 8.96 ± 4.0 N (Figure 7a). Accordingly, the brittle failure of the coating occurs at the penetration depth of about 5 μm which agrees with data shown in Figure 3. At the critical depth of penetration, the acoustic emission signal (AE) was detected, which shows coating failure. On the other hand, conformal cracking was observed for coatings oxidized at 700 °C and 800 °C. It should first be noted, that the high hardness of the as-deposited coating (37 GPa) decreases at temperature above 700 °C and consequently is fully delaminated at 1000 °C [18].

### 3.3. Examination of Oxidation-Based Notch Wear in Dry Machining Tests

As it was documented, the tool wear tests representing dynamic diffusion tests were carried out under the selected cutting speed of 200 m/min in dry turning conditions, to achieve the conditions similar to the oxidation tests. The chemical compositions of elements at points marked in Figure 8a and Figure 9a (SEM images for Inconel 718 and Ti6Al4V machining trials) are shown in Table 4. In points 3 in Figure 9a,b are the places at the tool-chip contacts where oxide layer Al_2_O_3_ occurs.

Figure 10 presents the SEM micrographs of flank faces observed after 2 min machining trials of IN 718 and Ti6-4 superalloys with visible wear patterns within the oxidized areas resulting from diffusion of ambient atmospheric air. The measured width of the notch wear is equal to about VB_N_ = 0.2 mm for the machining of these two superalloys. The chemical components of O, Al and Ti elements are similar to the initial case presented in Figure 8 and Figure 9. Similar tool wear patterns were observed in finishing turning of Inconel 718 by Cantero et al. [19].

**Figure 11:**
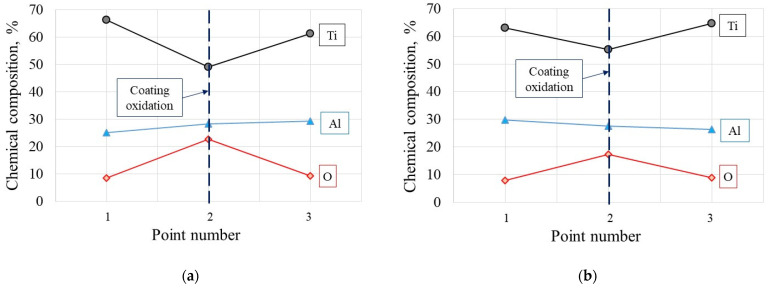
Average semi-quantitative chemical composition detected in points 1, 2 and 3 at the flank faces in Figure 8b and Figure 9b after machining of IN718 (**a**) and Ti6-4 (**b**) alloys with the cutting speed of 200 m/min; machining time of 30 s (data specified in Table 5).

**Figure 12:**
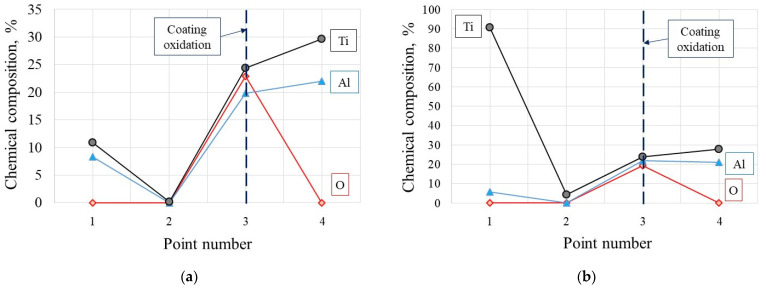
Average semi-quantitative chemical composition detected in points 1, 2 and 3 at the rake faces in Figure 8a and Figure 9a after machining of IN718 (**a**) and Ti6-4 (**b**) alloys with the cutting speed of 200 m/min; machining time of 30 s (data specified in Table 6, respectively).

**Figure 13:**
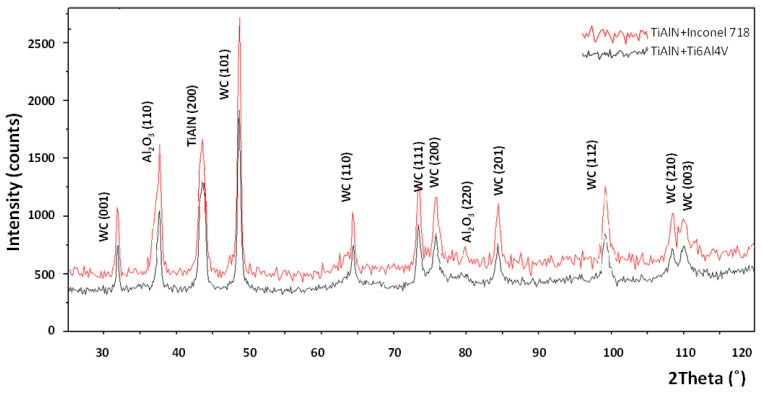
XRD analysis results of investigated coating after oxidation during machining tests.

It was shown that the Al_2_O_3_ layer is growing on the flank and rake faces (Figure 8 and Figure 9) and in the area of the notch wear presented in Figure 10. As shown in Table 4 and in Figure 11 and Figure 12, the conditions for the diffusion occurring in short machining trials of Inconel 718 and Ti6Al4V alloys are comparable. The content of the oxygen and aluminum is almost the same, but at the flank face the content of Ti is noticeably higher. Furthermore, they are similar to the EDS analysis made for the both alloys oxidized for a longer (approx. 30 min) time. Another finding is that small contents of O (about 5 at.%) and Al (about 8.5 at.%) were transferred onto the smeared workpiece (point 1 in Figure 9a). In addition, the data given in Table 3 shows that the content of oxygen and aluminum are almost the same. The crystallographic orientation of the Al_2_O_3_ ((220) at 2θ = 80°) and ((110) at 2θ = 38°) in Figure 13 are the same as shown in Figure 5 and Figure 6. However, a high content (25 at.% and 50–55 at%) of Ti was shown for flank and rake faces; it can confirm the presence of the TiAlN phase.

**Figure 14:**
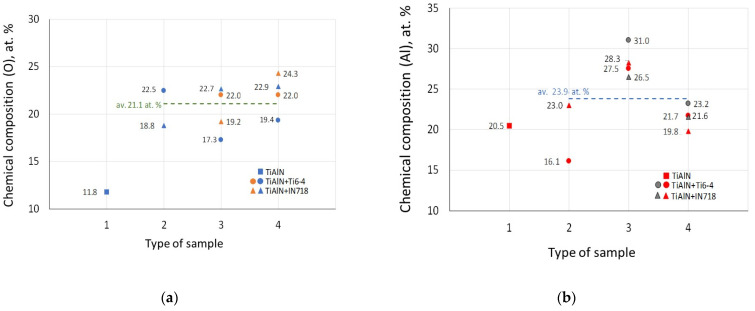
Comparison of oxidation conditions based on the at.% O (**a**) and Al (**b**) for different samples: 1, TiAlN; 2, diffusion couples; 3 and 4, rake and flank faces after machining with machining time of 0.5 min and 2 min (data specified in Table 2, Table 3 and Table 5, respectively).

Figure 14 presents the comparison of oxidation data collected for the four cases involved in this study. As mentioned previously, the main research strategy applied is to find the oxidation conditions based on the static diffusion tests which would be mostly similar to real machining operations and termed as dynamic tests. As can be seen in Figure 14a,b, the tests based on an oxidation in air provide decisively lower at.% for oxygen content but satisfactory at.% for Al content. However, the predictions based on the diffusion couples with the normal load of 150 N seem to be acceptable for the two material couples selected, namely, TiAlN + IN718 and TiAlN + Ti6-4 (see imbedded average lines). In particular, it is more accurate for the oxidation occurring at the flank face for both IN718 and Ti6-4 alloys.

**Figure 15:**
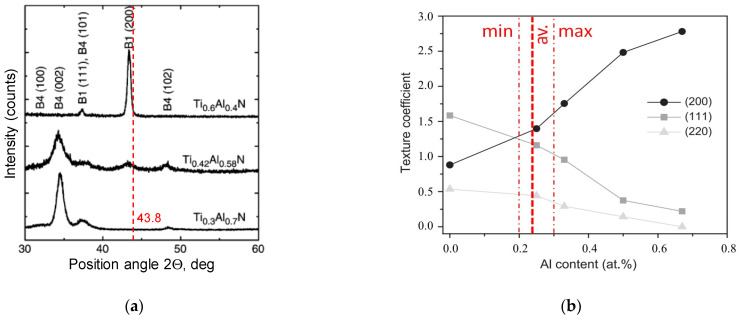
XRD patterns of TiAlN coatings with various atomic ratios (Ti:Al) (**a**) and the influence of Al at.% content on Ti1-xAlxN coating texture (**b**) with experimental data presented in Figure 13a,b (diagrams were elaborated based on Refs. [18,19]).

Figure 15a shows XRD patterns for three TiAlN coatings with various (Ti:Al) atomic ratios and compositions of Ti_0.6_A_l0.4_N, Ti_0.42_Al_0.58_N and Ti_0.3_Al_0.7_N which have a cubic (B1) structure and B1(200) preferred orientation, and a hexagonal B4 structure and B4 (002) preferred orientation, respectively. The TiAlN film is transformed from the B1 structure into the B4 structure when the Al content increases.

Figure 15b confirms that under oxidation conditions produced in the static and dynamic oxidation tests both crystallographic orientations (220) and (111) are likely to occur, which is documented by appropriate XRD diagrams shown in Figure 6 and Figure 13. The preferred crystallographic orientation (200) was detected for the angle 2θ equal to 43.8° for TiAlN coatings tested on the three diffraction patterns presented in Figure 5, Figure 6 and Figure 13 and comparatively in Figure 15a. On the other hand, under oxidation in air, different crystallographic orientations occur, such as (104) and (113), as presented in Figure 5. These facts suggest that the diffusion couples are more suitable for prediction of oxidation effects generated in machining operations under similar thermo-mechanical contact conditions. The oxidized coating surfaces can also be examined using Raman spectroscopy [20]. It was documented that wear debris produced on the coating surfaces can be characterized by different Raman active modes.

It can be concluded that future research in this area should be directed to find a stronger correlation of the oxidation rates under two oxygen access modes, namely, in static and dynamic diffusion tests, in order to elaborate a map showing the distribution of O and Al elements for the contact conditions suitable for the machining practice.

## 4. Conclusions

1. An original methodology for predicting oxidation effects for a TiAlN coated tool based on special diffusion couple tests under defined temperature regimes is proposed.
2. It was proven, based on EDS and XRD analyses, that the oxidation of TiAlN coatings in the static diffusion couples and dynamic machining tests provide comparable data when the Al_2_O_3_ layer is formed at the 900 °C.
3. The thickness of the oxide layer and the coating failure type can be predicted based on monitoring of the friction force and penetration depth during scratch tests.
4. It was revealed that the oxidation intensity depends on the coupled materials. In particular, higher mass changes and corresponding thicknesses of the oxidized layer were detected for TiAlN + Ti6Al4V couple versus a TiAlN + IN718 couple resulting from additional diffusion of Ti (30 at.% vs. 15 at.%).
5. Dynamic machining tests showed that oxidation of TiAlN coating deposited on the flank face occur which results in the creation of notch wear in the peripheral place with an easy access of ambient atmospheric air.
6. The comparison of chemical compositions and produced phases seems to be a decisive argument for the similarity of oxidized Al_2_O_3_ layers formed in static diffusion and dynamic machining. The consequence of intensive oxidation of the flank face is the evolution of notch wear.
7. Special maps showing the distribution of oxygen (O) and aluminum (Al) in the oxidized areas are plotted in order to simplify the comparison of the results of static and dynamic diffusion tests.

## Figures and Tables

**Table 1:** The estimated chemical composition of the contacted materials.

Materials	Chemical Composition, at.%									
	Ti	Al	N	V	Ni	Cr	Fe	Nb	Mo	Mn
TiAlN	27.1	21.4	51.5	-	-	-	-	-	-	-
Ti6Al4V	90.8	5.8	-	3.5	-	-		-	-	-
Inconel	1.1	-	-	-	54.9	21.1	16.5	3.3	2.9	0.4

**Table 2:** The estimated chemical composition of oxidized layers deposited during oxidation tests of TiAlN coating.

Element	Annealing Temperature			
	700 °C	800 °C	900 °C	1000 °C
O	18.1	18.9	23.1	28.3
Al	18.1	18.4	18.1	17.5
N	50.3	49.2	42.6	35.1
Ti	11.0	11.0	12.7	14.4

**Table 3:** The estimated chemical composition of oxidized layers deposited at 900 °C (presented graphically in Figure 4).

Element	Oxidized Sample								
	TiAlN			TiAlN + IN718			TiAlN + Ti6-4		
	2.5	10	30	2.5	10	30	2.5	10	30
O	9.3	11.8	17.9	17.1	18.8	20.3	21.0	22.4	24.6
Al	21.0	20.5	19.0	23.1	23.0	22.1	16.1	16.1	15.3
N	49.1	47.7	44.0	35.2	33.7	31.3	33.8	32.0	30.0
Ti	20.5	20.0	19.0	12.2	12.0	11.1	29.1	29.5	30.1

**Table 4:** The estimated chemical composition of oxidized layer formed in machining tests on the rake and flank faces in point # 3 in Figure 8b and point #2 in Figure 9b (data for all points are specified in Table 5).

Element, at.%		Machining Variant	
		TiAlN vs. IN 718	TiAlN vs. Ti6Al4V
O	Rake face	22.9	19.4
	Flank face	17.3	22.7
Al	Rake face	19.8	21.7
	Flank face	28.3	27.5
Ti	Rake face	24.4	23.7
	Flank face	49.1	55.2

**Table 5:** **a:** (**a**) EDS analysis results in points 1, 2 and 3 on the flank face marked in Figure 8b and Figure 9b. TiAlN vs. IN718; (**b**) TiAlN vs. Ti6Al4V. **b**: EDS analysis results in points 1, 2 and 3 on the rake face marked in Figure 8a and Figure 9a. (**a**) TiAlN vs. IN 718; (**b**) TiAlN vs. Ti6Al4V.

**a**							
**Point**	**Element (at.%)**			**Point**	**Element (at.%)**		
	**O**	**Al**	**Ti**		**O**	**Al**	**Ti**
x 1	8.5	25.1	66.4	x 1	7.9	29.7	63.0
x 2	22.7	28.3	49.1	x 2	17.3	27.5	55.2
x 3	9.4	29.3	61.3	x 3	8.9	26.3	64.8
**(a)**				**(b)**			
**b**							
**Point**	**Element (at.%)**			**Point**	**Element (at.%)**		
	**O**	**Al**	**Ti**		**O**	**Al**	**Ti**
x 1	4.8	8.3	10.9	x 1	-	5.6	90.7
x 2	-	-	0.2	x 2	-	-	4.3
x 3	22.9	19.8	24.4	x 3	19.3	21.7	23.7
x 4	-	22.0	29.7	x 4	-	21.0	27.7
**(a)**				**(b)**			

**Table 6:** **a.** At.% for oxidized layer produced in TiAlN + IN 718 dynamic diffusion couple at different points of the rake face in Figure 8a. Note that in point 1 the deposited workpiece material is detected, whereas in point 2 the WC-Co substrate is detected. On the other hand, in point 3 a fragment of Al_2_O_3_ oxidized layer is detected. In point 4, the oxygen is not present, which suggest the deposited Ti_29.7_Al_22_N_48.3_ coating. **b.** At.% for oxidized layer produced in TiAlN + Ti6Al4V dynamic diffusion couple at different points of the rake face in Figure 9a. Note that in point 1 the deposited Ti6Al4V (Ti_90.7_Al_5.6_V_3.8_) workpiece material is detected, whereas in point2 the WC-Co substrate is detected. On the other hand, in point 3 a fragment of Al_2_O_3_ oxidized layer is detected. In point 4, the oxygen is not present, which suggests the deposited Ti_27.7_ Al_21_N_51.3_ coating.

**a**									
Place	Element (at.%)								
	Co	W	Fe	O	Al	N	Ti	Ni	Cr
Point x 1	-	-	-	4.8	8.3	4.9	10.9	50.0	21.1
Point x 2	17.7	71.3	1.7	-	-	-	0.2	5.7	3.4
Point x 3	-	-	-	22.9	19.8	32.9	24.4	-	-
Point x 4	-	-	-	-	22.0	48.3	29.7	-	-
**b**									
Place	Element (at.%)								
	O	Al	N	Ti	V	Co	W	Fe	
Point x 1	-	5.6	-	90.7	3.8	-	-	-	
Point x 2	-	-	-	4.3	-	22.9	69.1	2.7	
Point x 3	19.4	21.7	35.2	23.7	-	-	-	-	
Point x 4	-	21.0	51.3	27.7	-	-	-	-	

## Data Availability

The data presented in this study are available on request from the corresponding author.
